# Glycyrrhizin Suppresses the Growth of Human NSCLC Cell Line HCC827 by Downregulating HMGB1 Level

**DOI:** 10.1155/2018/6916797

**Published:** 2018-01-15

**Authors:** Xiaojin Wu, Weitao Wang, Yuanyuan Chen, Xiangqun Liu, Jindong Wang, Xiaobin Qin, Dawei Yuan, Tao Yu, Guangxia Chen, Yanyan Mi, Jie Mou, Jinpeng Cui, Ankang Hu, Yunxiang E, Dongsheng Pei

**Affiliations:** ^1^Department of Radiation Oncology, The First People's Hospital of Xuzhou, Xuzhou, Jiangsu 221002, China; ^2^Genesis Beijing Co., Ltd., Beijing 100102, China; ^3^Department of Respiratory Diseases, The First People's Hospital of Xuzhou, Xuzhou, Jiangsu 221002, China; ^4^Department of Chest Surgery, The First People's Hospital of Xuzhou, Xuzhou, Jiangsu 221002, China; ^5^Department of Tumor, The First People's Hospital of Xuzhou, Xuzhou, Jiangsu 221002, China; ^6^Department of Gastroenterology, The First People's Hospital of Xuzhou, Xuzhou, Jiangsu 221002, China; ^7^Department of Pharmacy, Xuzhou Medical College, Xuzhou, Jiangsu 221004, China; ^8^Clinical Laboratory of Yantaishan Hospital, No. 91, Jiefang Road, Yantai, Shandong 264001, China; ^9^Laboratory Animal Center, Xuzhou Medical College, Xuzhou, Jiangsu 221004, China; ^10^Department of Pathology, Xuzhou Medical College, Xuzhou, Jiangsu 221004, China

## Abstract

Lung cancer has very high mortality and glycyrrhizin was found to significantly inhibit the growth of lung cancer cells* in vitro* and tissues in mice. However, the detailed inhibitory role of glycyrrhizin in the growth of lung cancer is still unclear. In this study, we first found that glycyrrhizin inhibited the growth of lung tumor in PDX mice. And high level of HMGB1 promoted the migration and invasion of lung cancer cells, which was suppressed by glycyrrhizin. Moreover, glycyrrhizin reduced the activity of JAK/STAT signaling pathway, which is the upstream regulator of HMGB1. Therefore, this study revealed a potential mechanism by which glycyrrhizin can inhibit the progression of lung cancer.

## 1. Introduction

Lung cancer is highly prevalent worldwide. An estimated 526,510 men and women living in the United States had a history of lung cancer in 2016 [1]. Traditionally, lung cancer can be divided into two types: small cell lung cancer (10–15%) and non-small cell lung cancer (NSCLC) (85–90%). The 5-year survival for NSCLC (21%) is usually higher than small cell lung cancer (7%). However, the 5-year relative survival rate of lung cancer is generally lower (17%) than other cancer types [1, 2]. Therefore, it is necessary to design novel drugs for effective treatment of lung cancer.

Glycyrrhizin, a glycoconjugated triterpene, is extracted from the roots of licorice plant,* Glycyrrhiza glabra*. It was first identified as an antiviral drug [3] and has been subsequently used in the treatment of patients with chronic hepatitis B and hepatitis C due to its anti-inflammatory role [4, 5]. Recently, glycyrrhizin was found to suppress lung adenocarcinoma A549 cell growth by inducing cancer cell apoptosis through downregulating the activity of thromboxane synthase pathway [6]. Furthermore, the growth of lung tumor tissue in PDX mouse model could be effectively inhibited by combining glycyrrhizin with cisplatin treatment, which showed low toxicity and side effects [6, 7]. Therefore, glycyrrhizin could be developed as a drug for lung cancer therapy.

High Mobility Group Box 1 (HMGB1) is a conserved nonhistone chromosomal protein that regulates nucleosome formation and gene transcription. HMGB1 could also be released into extracellular matrix, where it is recognized as a cytokine based on its role in mediating systemic inflammatory response [8]. Further, HMGB1 is involved in cancer progression and observed in various types of cancers [9–11]. High level of HMGB1 is always associated with cancer metastasis, suggesting that HMGB1 promotes invasion and metastasis of cancer cells [12–16]. Similarly, HMGB1 can promote the migration and invasion of lung cancer cells and regulate the metastasis of lung cancer [17, 18]. High serum level of HMGB1 can be a potential clinical biomarker for lung cancer [19–22].

Glycyrrhizin was found to inhibit HMGB1 for diagnosing HMGB1-mediated lethal systemic inflammation [23]. Glycyrrhizin could block the chemoattractant and mitogenic activities of HMGB1 by directly binding to its two HMG-box [24]. Moreover, glycyrrhizin could also regulate the expression of HMGB1 after hepatic ischemia-reperfusion (I/R) injury [25] and induction of traumatic pancreatitis in rats [26]. Moreover, glycyrrhizin can suppress tumor growth by reducing the level or activity of HMGB1 [27, 28]. However, whether the anti-lung cancer effect of glycyrrhizin involves suppression of HMGB1 remains unknown.

In this study, glycyrrhizin was found to suppress the tumor growth of NSCLC in PDX mice. Furthermore, the levels of both HMGB1 and its related JAK/STAT3 signal pathway factors were downregulated by treating PDX mice with glycyrrhizin. These findings indicated that glycyrrhizin may be involved in anticancer therapy of NSCLC through downregulating the level of HMGB1.

## 2. Materials and Methods

### 2.1. Cell Lines

Human NSCLC cell line HCC827 was purchased from the cell bank of Chinese Academy of Sciences and cultured in RPMI 1640 medium containing 10% fetal bovine serum (FBS).

### 2.2. PDX Mice Model

The logarithmic growth phase of HCC827 cells was suspended in cell culture medium and the concentration was adjusted to 1 × 10^7^ ml^−1^. Then, 0.2 ml cell suspension was subcutaneously injected into the back of nude mice, and the tumor formation was visually observed after 5 d. When the tumor grew to a diameter of 5–7 mm, 30 mice were randomly divided into the model group and glycyrrhizic acid treatment group, with 15 rats/group.

### 2.3. Glycyrrhizin Treatment

Glycyrrhizic acid (purchased from Dalian Meilen Pharmaceutical Technology Development Co., Ltd.) was diluted in DMSO and intraperitoneally injected at a dose of 100 mg/kg for two days, followed by continuous administration for two weeks. The model control group was injected with the same amount of DMSO.

### 2.4. Hematoxylin and Eosin Staining

Lung cancer cells from the glycyrrhizin treated and DMSO treated groups were stained by Hematoxylin and Eosin (H&E), as previously described [29]. First, samples were treated with distilled water and the nuclei were stained with the alum haematoxylin. Then they differentiated with 0.3% acid alcohol and were stained with eosin. Finally, they were dehydrated through 95% alcohol, cleared in 2 changes of xylene, and mounted with xylene based mounting medium.

### 2.5. Immunohistochemical Staining

The immunohistochemical staining was performed using EnvisionTM, as previously described [30]. Lung cancer tissue staining results were evaluated by IRS score under double-blind conditions by two senior pathological experts. The average staining score was calculated by combining the positive staining intensity and the percentage of positive cells.

### 2.6. Western Blot Analysis

Western blot analysis was performed as previously described [31]. Cells were lysed using RIPA lysis buffer and then proteins were extracted. Equal amounts of proteins were denatured in boiling SDS sample buffer and subjected to 10% SDS-PAGE. Then, the proteins were transblotted onto polyvinylidene difluoride membranes with a wet blot system. The membranes were blocked by 5% dry skim milk and incubated with primary anti-HMGB1 (Abcam), anti-P-Jak2, P-Stat3, Jak2, and Stat3 (Cell Signaling) antibodies. *β*-Actin was used as an internal control. Finally, the membranes were treated with enhanced chemiluminescent system for visualization of the protein bands. The bands were quantified using Image J software.

## 3. Results

### 3.1. Establishment of the PDX Mouse Model for Non-Small Cell Lung Cancer

**Figure 1:**
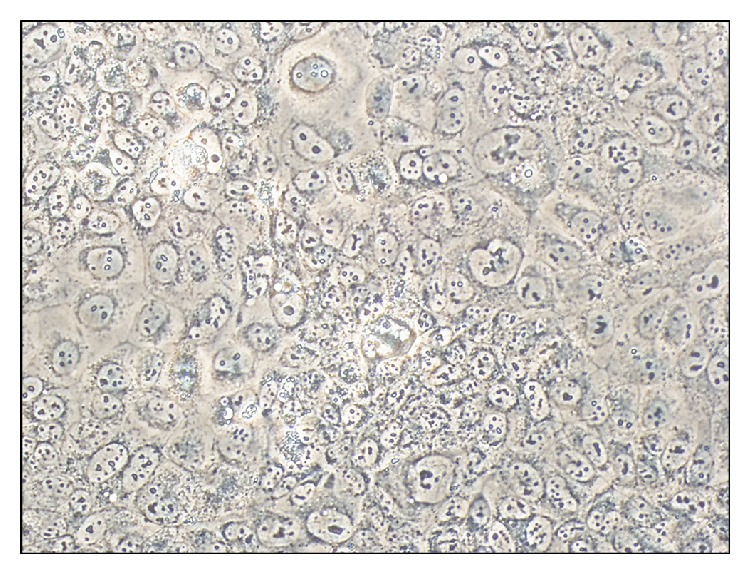
Morphology of lung cancer HCC827 cells in PDX mice.

For testing the effect of glycyrrhizin on the growth of NSCLC, we established the PDX mouse model using the lung cancer HCC827 cell line. Then, we observed the morphology of the cancer cells in PDX mice and found that the arrangement of cells and shape of nuclei were irregular (Figure 1).

### 3.2. Glycyrrhizin Inhibits Tumor Growth in the PDX Mouse Model

**Figure 2:**
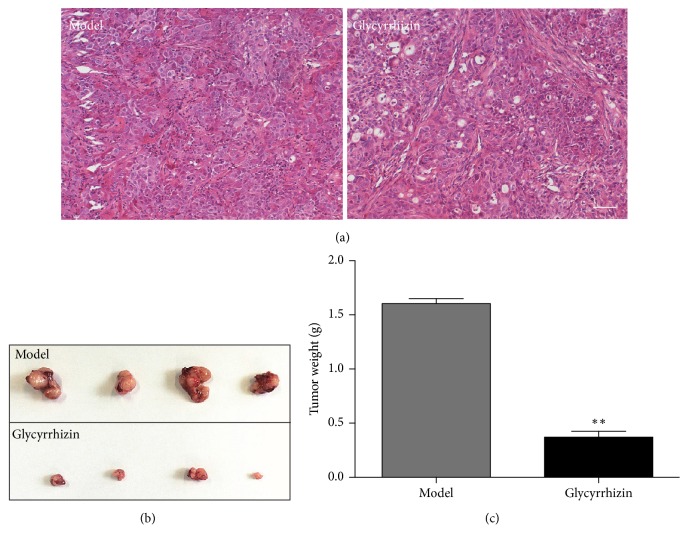
Glycyrrhizin inhibits tumor growth in PDX mice. (a) indicates the morphology of lung cancer cells from the two groups by HE staining, bar = 100 *μ*m. (b) indicates the size of tumors from the two groups. Tumors were excised from the two groups and the tumor sizes between the two groups were compared. (c) indicates the average weight of tumors in the two groups. The difference of tumor weights between the glycyrrhizin treatment and DMSO treatment (model) was significant. ^*∗∗*^*p* < 0.01, as compared to the model control by* t*-test.

We detected the effect of glycyrrhizin on tumor growth in PDX mice. The PDX mice were divided into two groups: glycyrrhizin treated and DMSO treated. We first observed the morphology of cancer cells by HE staining (Figure 2(a)). Next, we evaluated the tumor sizes and weights in the two groups. The glycyrrhizin treated mice had smaller tumor sizes than the DMSO treated mice (Figure 2(b)). The average tumor weight of the glycyrrhizin treated mice was significantly lower than the DMSO treated group (*p* < 0.01) (Figure 2(c)). These results suggested that glycyrrhizin can inhibit the growth of lung tumor in PDX mice.

### 3.3. HMGB1 Protein Is Suppressed by Glycyrrhizin

**Figure 3:**
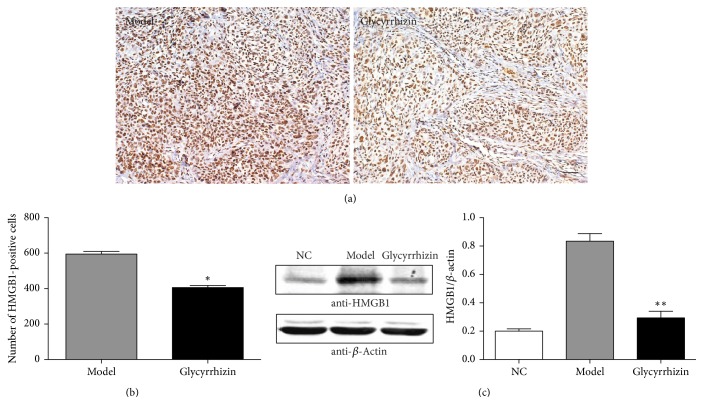
The protein level of HMGB1 is suppressed by glycyrrhizin. (a) indicates the protein level of HMGB1 in lung tumor tissues from the two groups detected by IHC staining assay, bar = 100 *μ*m. (b) indicates the quantitative results based on IHC staining assay. HMGB1-positive cells were counted in the two groups, respectively. ^*∗*^*p* < 0.05, as compared to the model control by* t*-test. (c) indicates the protein level of HMGB1 from the three groups by western blot. *β*-Actin was used as an internal control. *p* value was calculated by* t*-test between glycyrrhizin treatment and DMSO treatment (model). “NC” represents lung tissue from normal mice. ^*∗∗*^*p* < 0.01, as compared to the model control.

HMGB1 was reported to promote the migration and invasion of lung cancer cells and facilitate lung cancer metastasis [17, 18]. Glycyrrhizin functions as an inhibitor of HMGB1 by blocking its activity [24] or downregulating its expression [25]. However, whether the anticancer effect of glycyrrhizin in lung cancer relies on downregulating the expression of HMGB1 is unclear. So we detected the protein expression of HMGB1 in lung cancer tissues obtained from the two groups by IHC staining, and representative images are shown in Figure 3(a). Furthermore, the protein level of HMGB1 in the glycyrrhizin treated group was significantly lower than that in the DMSO treated group (*p* < 0.05) (Figure 3(b)). We also detected the protein level of HMGB1 by western blot and observed similar results. Interestingly, higher level of HMGB1 was also seen in lung tumor tissue from PDX mice compared to that from normal mice (NC), which is consistent with previous reports indicating that HMGB1 is related to cancer progression. However, the level of HMGB1 obviously decreased after glycyrrhizin treatment (Figure 3(c)), suggesting that HMGB1 protein is suppressed by glycyrrhizin.

### 3.4. Glycyrrhizin Inhibits the Phosphorylation of Jak2 and Stat3

In mammals, the JAK/STAT pathway is the principal signaling mechanism of inflammation and involves various cytokines and growth factors [32, 33]. Members of the JAK family are receptor-associated tyrosine kinases activated by various extracellular signals. Signal Transducer and Activator of Transcription (STAT) proteins are the typical substrates of JAK kinases and are generally associated with transcriptional activation as transcription factors [34]. HMGB1 is released from the nucleus into the cytoplasm, which is regulated by JAK/STAT signal pathway mediated HMGB1 hyperacetylation [35]. Furthermore, resveratrol could reduce the release of HMGB1 from the nucleus to the cytoplasm by suppressing the activity of STAT signaling pathway [36].

**Figure 4:**
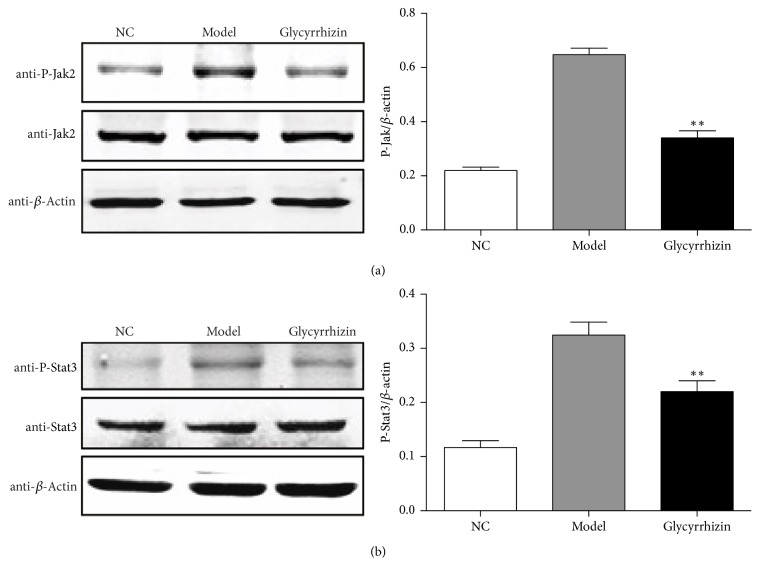
Glycyrrhizin inhibits the phosphorylation of Jak2 and Stat3. The phosphorylation level of Jak2 was detected by western blot in the three experimental groups, respectively. ^*∗∗*^*p* < 0.01, as compared to the model control (a). The phosphorylation level of Stat3 was detected by western blot in the three experimental groups, respectively. ^*∗∗*^*p* < 0.01, as compared to the model control (b). *β*-Actin was used as an internal control. *p* value was calculated by* t*-test between glycyrrhizin treatment and DMSO treatment (model). “NC” represents lung tissue from normal mice. Stars are showing *p* values between model and glycyrrhizin.

We further examined whether glycyrrhizin could inhibit the activity of JAK/STAT signaling pathway. The phosphorylation status of Jak2 and Stat3 was detected by specific phosphorylated antibodies, which showed that the phosphorylation levels of Jak2 and Stat3 were significantly higher in the PDX-model mice, but obviously lower after glycyrrhizin treatment (Figures 4(a) and 4(b)). These results indicated that glycyrrhizin can inhibit the activity of JAK/STAT signaling pathway, which is the upstream regulator of HMGB1.

## 4. Discussion

In this study, glycyrrhizin was shown to suppress the growth of lung tumor tissues in PDX mice, derived from NSCLC HCC827 cell line, which is consistent with recent reports on the anticancer effect of glycyrrhizin on lung cancer progression [6, 7]. Huang et al. [6] showed that glycyrrhizin could inhibit the lung adenocarcinoma A549 cell line growth both* in vitro *and in PDX mice. Subsequently, Deng et al. [7] reported that glycyrrhizin combined with cisplatin had a better anticancer effect in the PDX mice model. We used another NSCLC cell line for establishing the PDX mice and proved that anticancer effect of glycyrrhizin was similar. Our findings further confirmed that glycyrrhizin may be a potential anticancer drug for NSCLC.

HMGB1, a cytokine, has extracellular functions in inflammation and cancer progression. As a late modulator of inflammation, HMGB1 functions as a damage-associated molecular pattern in the sterile inflammation model by amplifying hepatic ischemia/reperfusion (I/R) and acetaminophen-induced liver necrotic injury [37, 38]. Glycyrrhizin can antagonize the inflammatory effect of HMGB1 by suppressing the expression of HMGB1 in hepatic I/R injury [25]. In addition, HMGB1 has a critical role in cancer metastasis. High levels of HMGB1 are always observed in various cancer types, including ovarian [15], liver [37], and lung [22] cancers. HMGB1 can promote the migration and invasion of lung cancer cells [18]. Drugs designed for regulating the level of HMGB1 may have a potential clinical value for lung cancer patients. In this study, high level of HMGB1 was related to the growth of lung tumors in PDX mice. Glycyrrhizin obviously inhibited the level of HMGB1. Therefore, glycyrrhizin acts as an inhibitor of HMGB1 and the growth of lung tumor. The direct role of HMGB1 in anticancer effect of glycyrrhizin on lung cancer progression needs further study.

Glycyrrhizin influenced the upstream regulator of HMGB1, the JAK/STAT signaling pathway. HMGB1 release from nucleus to cytoplasm is regulated by the activation of JAK/STAT signaling pathway [35]. We found that glycyrrhizin can block the activity of JAK/STAT signaling pathway by inhibiting phosphorylation of Jak2 and Stat3. This is the first study to show that the level of HMGB1 may be controlled by the JAK/STAT signaling pathway.

## 5. Conclusion

Glycyrrhizin inhibits the growth of lung tumors in PDX mice by downregulating the level of HMGB1. This mechanism is potentially due to the inhibition of JAK/STAT signaling pathway by glycyrrhizin. Glycyrrhizin may be further investigated as a potential drug for NSCLC.
